# Under pressure: the interplay of hypertension and white matter hyperintensities with cognition in chronic stroke aphasia

**DOI:** 10.1093/braincomms/fcae200

**Published:** 2024-06-11

**Authors:** Jade Hannan, Natalie Busby, Rebecca Roth, Janina Wilmskoetter, Roger Newman-Norlund, Chris Rorden, Leonardo Bonilha, Julius Fridriksson

**Affiliations:** Department of Communication Sciences and Disorders, University of South Carolina, Columbia, SC 29208, USA; Department of Communication Sciences and Disorders, University of South Carolina, Columbia, SC 29208, USA; Department of Neurology, Emory University, Atlanta, GA 30322, USA; Department of Health and Rehabilitation Sciences, Medical University of South Carolina, Charleston, SC 29425, USA; Department of Psychology, University of South Carolina, Columbia, SC 29208, USA; Department of Psychology, University of South Carolina, Columbia, SC 29208, USA; Department of Pharmacology, Physiology, and Neuroscience, University of South Carolina School of Medicine, Columbia, SC 29209, USA; Department of Communication Sciences and Disorders, University of South Carolina, Columbia, SC 29208, USA

**Keywords:** brain health, hypertension, aphasia, stroke, cognition

## Abstract

While converging research suggests that increased white matter hyperintensity load is associated with poorer cognition, and the presence of hypertension is associated with increased white matter hyperintensity load, the relationship among hypertension, cognition and white matter hyperintensities is not well understood. We sought to determine the effect of white matter hyperintensity burden on the relationship between hypertension and cognition in individuals with post-stroke aphasia, with the hypothesis that white matter hyperintensity load moderates the relationship between history of hypertension and cognitive function. Health history, Fazekas scores for white matter hyperintensities and Wechsler Adult Intelligence Scale Matrix Reasoning subtest scores for 79 people with aphasia collected as part of the Predicting Outcomes of Language Rehabilitation study at the Center for the Study of Aphasia Recovery at the University of South Carolina and the Medical University of South Carolina were analysed retrospectively. We found that participants with a history of hypertension had increased deep white matter hyperintensity severity (*P* < 0.001), but not periventricular white matter hyperintensity severity (*P* = 0.116). Moderation analysis revealed that deep white matter hyperintensity load moderates the relationship between high blood pressure and Wechsler Adult Intelligence Scale scores when controlling for age, education, aphasia severity and lesion volume. The interaction is significant, showing that a history of high blood pressure and severe deep white matter hyperintensities together are associated with poorer Matrix Reasoning scores. The overall model explains 41.85% of the overall variation in Matrix Reasoning score in this group of participants. These findings underscore the importance of considering cardiovascular risk factors in aphasia treatment, specifically hypertension and its relationship to brain health in post-stroke cognitive function.

## Teaser

Hannan *et al*. report that deep white matter hyperintensity severity moderates the effect of hypertension on cognitive performance in individuals with chronic post-stroke aphasia. These findings underscore the importance of cardiovascular risk factors in aphasia recovery, specifically hypertension and its relationship to brain health in post-stroke cognitive function.

## Graphical Abstract

**Graphical abstract:**
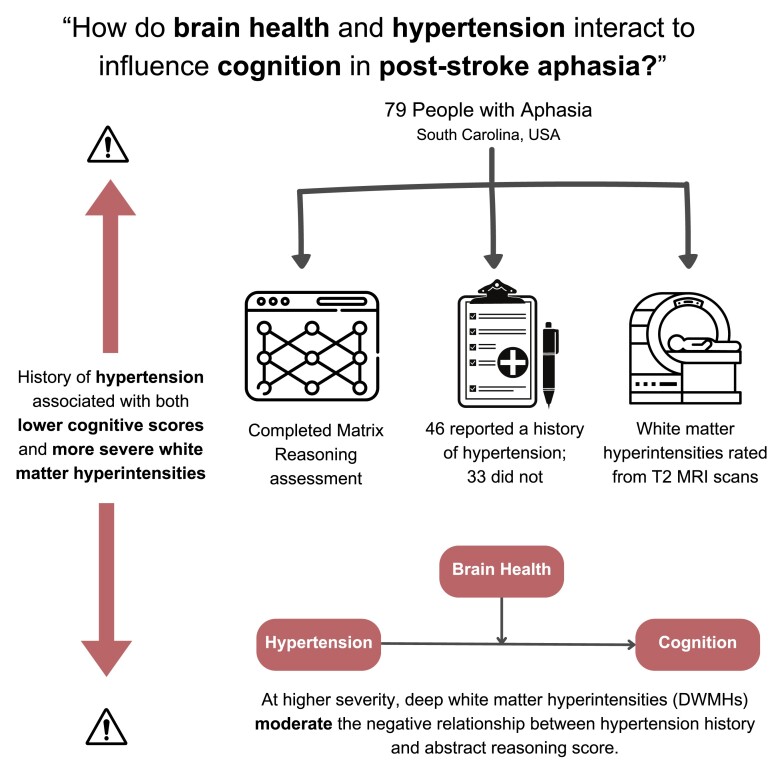

## Introduction

In recent years, the incidence of stroke in younger individuals has increased, whereby approximately 22% of stroke survivors are aged between 15 and 49 years.^1^ This means that individuals are living longer with chronic post-stroke symptoms such as aphasia, and there is a higher likelihood of co-occurring diseases developing which may impact stroke recovery. Indeed, more than one in five people develop dementia following a stroke,^2^ and in individuals with aphasia, recovery in the chronic stage regresses in approximately 30% of individuals.^3,4^

Recent work has demonstrated an association between chronic aphasia declines and critical modifiable and unmodifiable risk factors such as lesion volume, age at stroke and health factors.^4-7^ Many of the risk factors associated with long-term declines in chronic aphasia have also been identified as risk factors for dementia.

One of these modifiable risk factors is hypertension, characterized by abnormally elevated systolic and/or diastolic blood pressure. Hypertension is an increasingly prevalent condition, particularly among adults over the age of 60.^8^ Globally, an estimated 1.4 billion individuals have hypertension, which is often undiagnosed and/or uncontrolled.^9^ Hypertension can be managed through pharmacological treatment and lifestyle modifications; however, more research is needed to understand brain health risks associated with hypertension, particularly, in an ageing population. The relationship between hypertension, cerebrovascular accidents and cognitive decline (namely, vascular cognitive impairment and Alzheimer’s disease) is well-established, with hypertension described as the most important cerebrovascular risk factor leading to stroke and dementia, apart from age.^8,10,11^

Further, hypertension contributes to stroke risk and cognitive decline through the development of cerebral small vessel disease, markers of which include white matter hyperintensities (WMHs), enlargement of perivascular spaces, microbleeds, atrophy and lacunar infarcts, according to the STandards for ReportIng Vascular changes on nEuroimaging (STRIVE) guidelines.^8,12^ The most investigated markers of small vessel disease are WMHs, also called white matter lesions or leukoaraiosis. WMHs may be indicative of insufficient cerebrovascular supply, inflammation or blood–brain barrier leakage and present as hyperintense regions on T2-weighted and fluid-attenuated inversion recovery magnetic resonance imaging scans. While WMHs are typically more severe with the presence of hypertension, the precise aetiology of these hyperintensities is not well understood.^12-15^ The Fazekas scale is a visual scale commonly used to rate the severity of WMH on magnetic resonance imaging.^16^ A total WMH load score is calculated by summing two subscales, one of deep white matter hyperintensity (DWMH) severity and one of periventricular white matter hyperintensity (PVH) severity.

Importantly, two risk factors for increased WMH severity are age and hypertension, with a longitudinal cohort study finding an association between the presence of hypertension and increased risk of high WMH load.^8,13,17^ Both independently and as a component of comprehensive small vessel disease scores, WMHs are associated with cognitive impairment in healthy ageing.^18-21^ Similarly, in patients with a history of stroke, greater WMH severity is associated with poorer performance on cognitive tasks.^22-24^ Unsurprisingly, stroke patients tend to present with higher prevalence of cardiovascular risk factors (e.g. hypertension) than those who have not experienced a stroke.^22^

While converging research suggests that in ageing, increased WMH load is associated with poorer cognition, and the presence of hypertension is associated with increased WMH load, the interaction between hypertension, cognition and WMHs is not well understood. Given that individuals who have experienced a stroke typically have a higher prevalence of cardiovascular risk factors and may experience cognitive decline following a stroke, this interaction may be particularly important to understand. It is also possible that for individuals experiencing long-term consequences of stroke, such as aphasia, early signs of cognitive decline may be missed, therefore understanding this interaction could be critical. Therefore, we sought to investigate the implications of the interaction between hypertension and WMH severity for cognition in stroke survivors with aphasia, with the hypothesis that WMH severity moderates the relationship between history of hypertension and cognitive function in post-stroke aphasia.

## Materials and methods

### Participants

Data were obtained retrospectively from participants (*N* = 79) participating in the Predicting Outcomes of Language Rehabilitation (POLAR) clinical trial at the Center for the Study of Aphasia Recovery at the University of South Carolina and the Medical University of South Carolina. All participants gave informed consent for study participation in accordance with the Declaration of Helsinki. Behavioural testing took place at research laboratories at University of South Carolina and Medical University of South Carolina. American Speech-Language-Hearing Association (ASHA)-certified speech-language pathologists with experience working with individuals with aphasia administered all assessments.

The following inclusion/exclusion criteria were applied: Participants were included in the study if they (i) had experienced a left-hemisphere ischaemic or haemorrhagic stroke, (ii) had chronic aphasia (≥12 months post-stroke), (iii) were aged between 21 and 80 years, (iv) had spoken English as their primary language for at least 20 years and (v) were able to provide written or verbal consent. Participants were excluded if they had (i) severely limited verbal output, (ii) severely impaired auditory comprehension, (iii) bilateral or cerebellar stroke or (iv) contra-indications to testing with magnetic resonance imaging. Individuals with multiple strokes were eligible if all lesions were confined to the left supratentorial territory.

### Behavioural testing

As part of the Predicting Outcomes of Language Rehabilitation protocol, participants underwent extensive baseline language and neuropsychological testing at the time of enrolment (see Kristinsson *et al*. for detailed protocol).^25^ Language testing included the Western Aphasia Battery (WAB), from which an overall aphasia severity score [Aphasia Quotient (WAB AQ)] was calculated for each participant. Behavioural tests were administered on a laptop (MacBook Pro) or an iPad by ASHA-certified speech and language pathologists with experience working with individuals with aphasia. The behavioural testing included the Wechsler Adult Intelligence Scale (WAIS) Matrix Reasoning subtest,^26^ which assesses inductive reasoning and is often considered a measure of fluid intelligence.^26^ Measures of cognition which depend minimally on the faculty of language are considered preferable when working with aphasic populations to minimize confounding effects that could arise during tasks requiring verbal skills, where errors may reflect the presence of a language disorder rather than a distinct cognitive impairment.^7^

It should nonetheless be noted that, while the Matrix Reasoning subtest itself does not include a verbal component, instructions for WAIS subtests are provided verbally per standard protocol. In addition, a previous study by Dugbartey and colleagues^27^ reported a significant association between scores on the Matrix Reasoning subtest and verbal fluency among adults seen at a neuropsychiatry clinic, leading the authors to suggest that linguistically mediated problem-solving strategies may mediate performance on the Matrix Reasoning test despite its not requiring verbal production. The same study also found high correlations between Matrix Reasoning scores and performance on the Comprehensive Test of Nonverbal Intelligence.^27^ As such, language comprehension abilities may play a role in performance on this measure as with most formal cognitive measures. We attempted to address this potential confound by excluding individuals with severely impaired auditory comprehension and by controlling for lesion volume and aphasia severity (WAB AQ) in our analyses.

### Health history

Health factors, including history of high blood pressure, were self-reported in a health questionnaire, and responses were corroborated by medical records when available. Patients were asked if they had ever been diagnosed with high blood pressure (hypertension). As medication and current physiological data were not available for this retrospective study, we were not able to distinguish among current/active, medication-controlled and undiagnosed hypertension. From this point forward, the term ‘hypertension’ is used to refer to a known medical history of hypertension.

### Magnetic resonance imaging data acquisition and preprocessing

Patients underwent high-resolution T1- and T2-weighted neuroimaging on a Siemens Trio 3T scanner equipped with a 12-channel (Trio configuration) or 20-channel (following upfit to Prisma configuration) head coil using the following parameters: T1-weighted imaging utilized an MP-RAGE sequence with 1 mm isotropic voxels, a 256 × 256 matrix size, a 9° flip angle and a 92-slice sequence with repetition time = 2250 ms, inversion time = 925 ms and echo time = 4.11 ms. T2-weighted scans were acquired using the same angulation and volume centre as the T1 scan. This 3D T2-weighted SPACE sequence used a resolution of 1 mm^3^ with a field of view = 256 × 256 mm, 160 sagittal slices, variable degree flip angle, repetition time = 3200 ms, echo time = 212 ms and ×2 GeneRalized Autocalibrating Partial Parallel Acquisition (GRAPPA) acceleration (80 reference lines).

**Figure 1:**
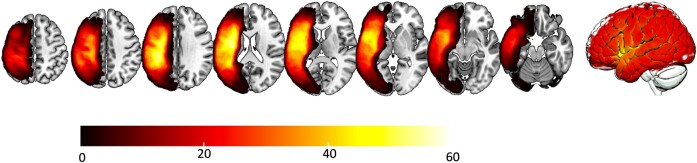
**Lesion overlay map across all participants.** Lesion overlay map showing overlap of stroke lesions for all participants.

Chronic stroke lesions were manually drawn onto each participant’s native-space T2-weighted image by a neurologist (author L.B.) or trained study staff member (author R.N.N), both of whom were blinded to participant demographics and behavioural data, see Fig. 1 for lesion overlay. Enantiomorphic normalization was conducted using the *nii_process­ pipeline* (https://github.com/neurolabusc/nii_preprocess), a set of Matlab-based (R2017b, The MathWorks) script that leverages multiple best-of-breed programs^28^ [SPM12: Functional Imaging Laboratory, Wellcome Trust Centre for Neuroimaging, Institute of Neurology (www.fil.ion.ucl.ac.uk/spm), FSL v6.0.3, ASLtbx (https://www.cfn.upenn.edu/zewang/ASLtbx.php) and MRItrix (https://www.mrtrix.org/)] in order to normalize and process magnetic resonance imaging data acquired from individuals with lesioned brains. These scripts utilized enantiomorphic normalization^29^ to create a mirrored image of the right hemisphere, which was co-registered with the native T1 image. A chimeric image (i.e. a ‘healed’ brain) was then created whereby the damaged portion of the left hemisphere was temporarily replaced with the mirror image of intact areas from the healthy right hemisphere.^29^ SPM12’s unified segmentation–normalization^30^ warped this chimeric image to standard space. Each T2 image was co-registered to the corresponding T1 image, and binary lesion maps were then spatially transformed into native T1 space using the normalization function calculated during the enantiomorphic segmentation–normalization procedure. This additional step (enantiomorphic normalization) ensures that segmentation–normalization methods designed for intact brains do not incorrectly warp scans with large lesions to the left hemisphere. The resulting spatially transformed lesion maps were smoothed with a 3 mm full-width half-maximum Gaussian kernel to remove sharp edges associated with hand drawing.

### Identifying white matter hyperintensities

**Figure 2:**
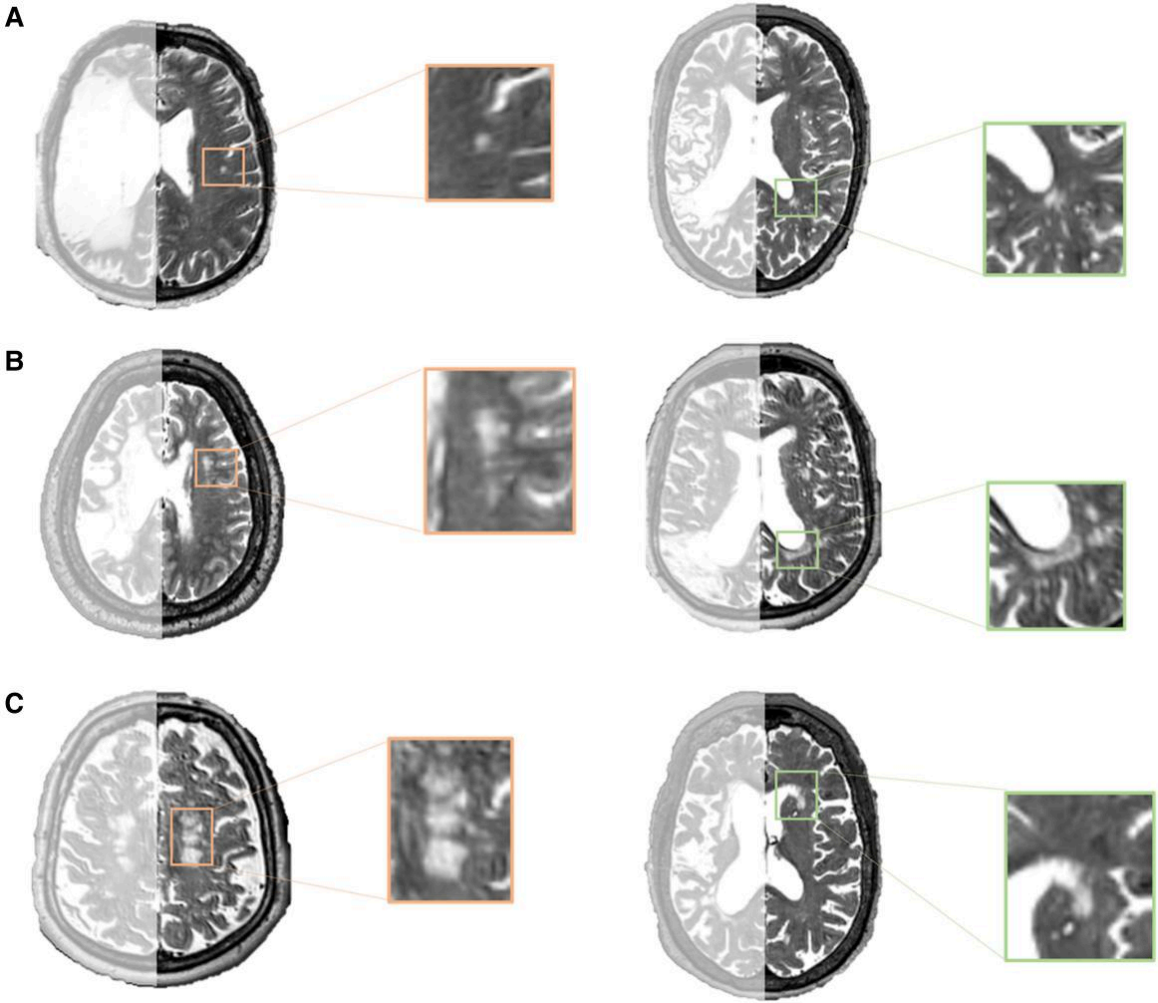
**Examples of WMHs.** Orange boxes (left column) show DWMHs, while green boxes (right column) highlight PVHs. **A** is an example of mild WMHs with a Fazekas score of 1 for PVHs and DWMHs. **B** shows moderate WMHs with a Fazekas score of 2 for PVHs and DWMHs. **C** is an example of more severe WMHs with a Fazekas score of 3 for PVHs and DWMHs.

The severity of WMHs was used as a measure of brain health. To avoid problems associated with the quantification of WMHs in the lesioned hemisphere, we chose to score WMHs in the intact, contralesional hemisphere only, as seen in previous studies involving participants with stroke.^23,31,32^ WMHs were identified based on raw T2-weighted scans using the Fazekas scale.^16^ The Fazekas scale includes separate ratings (0–3) for PVH and DWMH individually, and scores can be summed to give a total WMH severity score ranging from 0 to 6 (where higher scores indicate more severe WMHs), see Fig. 2 for example of different Fazekas ratings. WMHs were rated by two individuals (authors N.B. and R.R.), trained by a neurologist (author L.B.). Each scan was rated separately by the two raters, and inter-rater reliability was calculated for these initial ratings. Following this, any discrepancies were discussed until a consensus was reached for each scan. The consensus ratings were used in the final analyses.

### Statistical analysis

Cohen’s *k* was run to determine inter-rater reliability for WMH ratings. There was good agreement between the two raters’ scores for PVH [*k* = 0.730 (95% CI, 0.621–0.839)], DWMH [*k* = 0.814 (95% CI, 0.698–0.930)] and total Fazekas ratings [*k* = 0.703 (95% CI, 0.597–0.809)]. Note that after these initial ratings, raters discussed discrepancies until a consensus was reached, aided by a neurologist if necessary.

To explore differences between individuals with and without hypertension, we conducted independent t-tests with the following dependent variables: age, years of education, lesion volume, sex, months post-stroke and WAB AQ scores.

To establish the pairwise relationships between the variables, we used two separate Mann–Whitney U-tests (due to non-parametric data) to compare (i) WAIS scores in participants with and without hypertension and (ii) WMH severity in participants with and without hypertension. To investigate the relationship between WAIS scores and WMH severity, we used Kendall’s tau correlation analysis (for non-parametric data). We then used moderation analysis, controlling for age, lesion volume, WAB AQ and education to investigate the interaction effect of WMH severity on the relationship between hypertension and WAIS (Matrix Reasoning) scores. In the moderation analysis, WAIS score served as the dependent variable with the WMH score as the moderating variable and hypertension (binary yes/no) as the independent variable. Note that previous Mann–Whitney U-tests and Kendall’s tau correlations were run separately for DWMH and PVH. Subsequent moderation analyses were only run if these tests showed significant differences in WMH severity and history of hypertension and behaviour. Moderation analysis differs from mediation analysis in that a moderating variable, when significantly affecting the relationship between the independent and dependent variables, can enhance the association between the two by interacting with the independent variable. However, it is important to note that moderation analysis does not assert that the moderating variable is the underlying cause of the independent variable’s effect on the dependent variable outcome. Statistical analysis was conducted in SPSS 27 with the PROCESS toolbox.^33^

## Results

### Participants

Of the 107 people with aphasia who participated in the Predicting Outcomes of Language Rehabilitation trial, full neuroimaging, behavioural and medical history data were available for 79 people. Participants (*N* = 79 individuals; 47 males, 32 females) had a mean age of 56.92 years (*SD* = 11.12) at the time of their last stroke, and the mean age at the time of testing was 61.65 years (*SD* = 10.20). The majority of participants were non-Hispanic white (*n* = 57; 72%), 18 were Black or African American and 1 was Asian; race information was unavailable for 3 participants. At the time of cognitive assessment, participants were an average of 56.21 months post-stroke (*SD* = 57.24) and had an average WAB AQ of 60.87 (*SD* = 24.79). See Table 1 for full demographic information.

**Table 1:** Demographic information for participants

Demographic variables (*n* = 79)	Mean (SD)/count
Test age	61.65 (10.20)
Education (years)	15.72 (2.28)
Race (*n*)	White (57), African American (18), Asian (1), unknown/other (3)
Months post-stroke	56.21 (57.24)
Stroke age (from last stroke)	56.92 (11.12)
Fazekas score: DWMH	1.44 (0.89)
Fazekas score: PVH	1.73 (1.08)
Fazekas score: Total	3.19 (1.74)
Lesion volume (cubic centimetres)	126.42 (88.68)
Sex (males:females)	47:32
Hypertension (no hypertension:hypertension)	33:46
WAIS matrices scores (raw subtest score)	12.26 (5.82)
WAB AQ	60.87 (24.79)
Aphasia types (count)	
Anomia	23
Broca’s	40
Conduction	9
Global	3
Transcortical motor	1
Wernicke’s	3

### Participants with and without hypertension

Independent *t*-tests revealed no significant differences between individuals with and without hypertension for the following variables: age, years of education, lesion volume, sex, months post onset and WAB AQ, see Table 2.

**Table 2:** Demographic information for participants with and without hypertension

Variable	No hypertension (mean, SD)	Hypertension (mean, SD)	*t*	*P*
Age	59.36 (10.07)	63.28 (10.09)	−1.704	0.092
Years of education	15.88 (2.23)	15.61 (2.34)	0.515	0.608
Lesion volume (cm^3^)	133.66 (93.65)	121.23 (85.61)	0.612	0.543
Sex (males:females)	23:10	24:22	1.569	0.121
Months post onset	55.30 (54.67)	56.87 (59.60)	−0.119	0.905
WAB AQ	64.83 (22.72)	58.03 (26.05)	1.205	0.232
Total Fazekas score	2.57 (1.30)	3.65 (1.89)	−2.694	0.009
DWMH	1.03 (0.64)	1.74 (0.93)	−3.787	<0.001
PVH	1.50 (1.04)	1.90 (1.08)	−1.555	0.125

### Hypertension and Wechsler Adult Intelligence Scale scores

**Figure 3:**
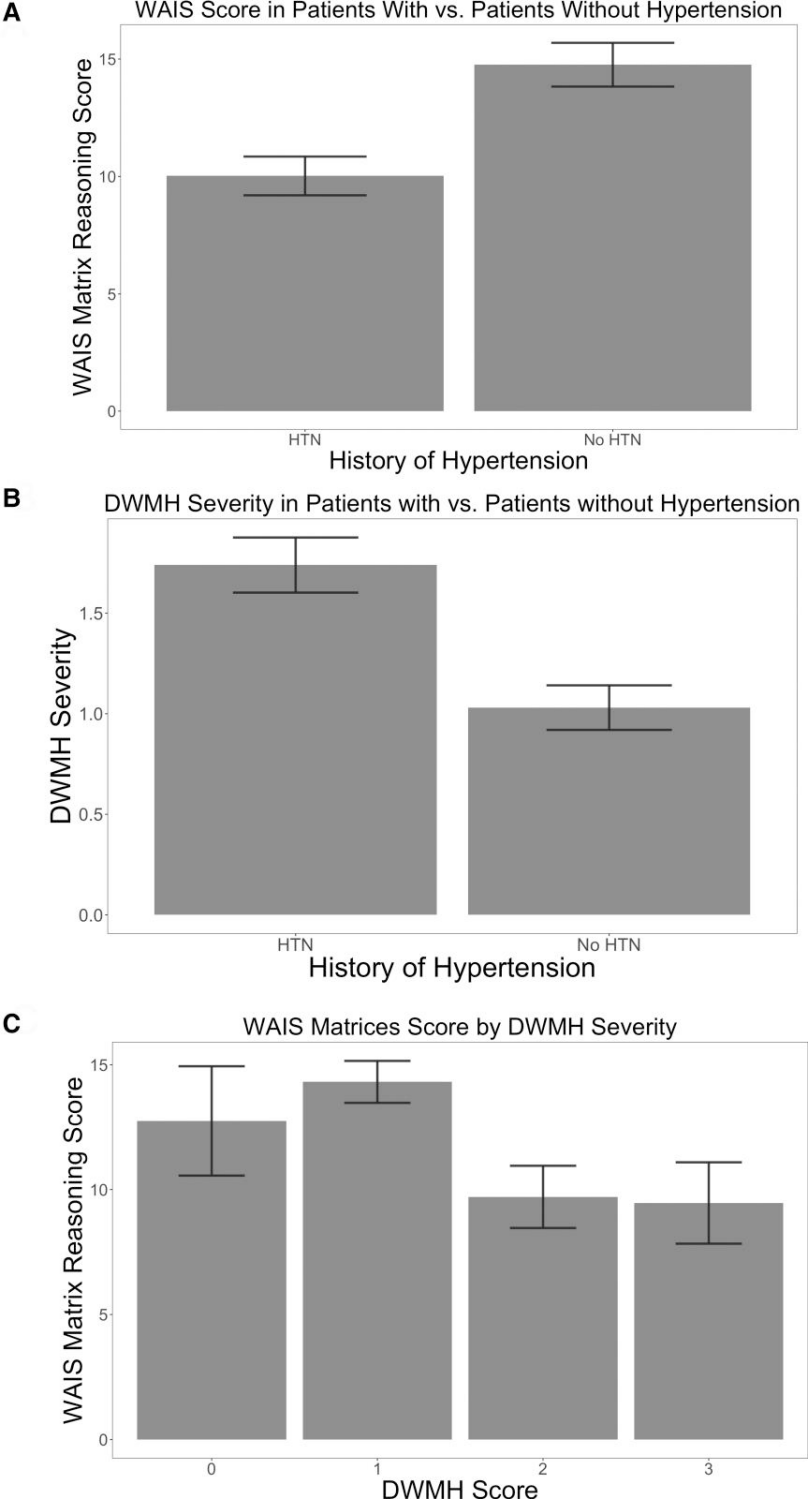
**Behavioural scores, DWMH and hypertension status in chronic stroke aphasia.** (**A**) Matrix Reasoning score by hypertension status in chronic stroke aphasia. Mann–Whitney U-tests were used to determine mean WAIS scores in participants with and without hypertension (with hypertension: *M* = 10.65, without hypertension: *M* = 14.76, *P* = 0.002). Error bars indicate standard error of the mean WAIS scores. (**B**) DWMH severity by hypertension status in chronic stroke aphasia. Mann–Whitney U-tests were used to determine mean DWMH severity in participants with and without hypertension (with hypertension: *M* = 1.74, without hypertension: *M* = 1.03, *P* < 0.001). Error bars indicate standard error of the mean DWMH severity. (**C**) Matrix Reasoning score by DWMH category in chronic stroke aphasia. Kendall’s tau correlation analysis was used to determine mean WAIS scores by DWMH severity (Fazekas score) (0: *M* = 12.75, 1: *M* = 14.32, 2: *M* = 9.71, 3: *M* = 9.46, *P* < 0.001). Error bars indicate the standard error of the mean WAIS scores.

Mann–Whitney U-tests revealed that individuals with hypertension had significantly reduced WAIS scores [severity (*M* = 10.65, *SD* = 5.59, *P* = 0.002)] compared with those without hypertension (*M* = 14.76, *SD* = 5.35), see Fig. 3A.

### White matter hyperintensity severity and hypertension

Individuals with hypertension had significantly higher Fazekas scores (*M* = 3.65, *SD* = 1.89, *P* = 0.010) compared with those without hypertension (*M* = 2.57, *SD* = 0.30). To identify whether the relationship between WMH severity and hypertension was driven by PVH or DWMH, we conducted separate Mann–Whitney U-tests. There was no significant difference in PVH severity (*P* = 0.116) for individuals with or without hypertension (hypertension: *M* = 1.90, *SD* = 1.08, without hypertension: *M* = 1.50, *SD* = 1.04). However, individuals with hypertension had significantly higher DWMH severity (*M* = 1.74, *SD* = 0.92, *P* < 0.001) compared with those without hypertension (*M* = 1.03, *SD* = 0.64), see Fig. 3B.

### White matter hyperintensity severity and Wechsler Adult Intelligence Scale

Kendall’s tau correlation analysis revealed a significant negative correlation between WMH severity and WAIS scores [*r*(79) = −0.211, *P* = 0.018]. To identify if this relationship was driven by PVH, DWMH or both, we conducted separate correlational analyses. These analyses revealed a significant negative correlation between WAIS scores and DWMH severity [*r*(79) = −0.265, *P* < 0.001] but not PVH severity [*r*(79) = −0.146, *P* = 0.146], see Fig. 3C.

### Moderation analysis

**Figure 4:**
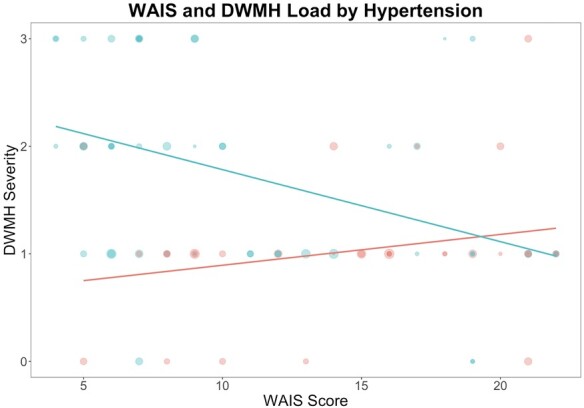
**Moderating effect of DWMH severity on the relationship between history of hypertension and WAIS score.** DWMH severity is reported as the deep WMH Fazekas score for each participant. Red dots represent participants without hypertension; blue dots represent patients with hypertension. Dot size corresponds to lesion volume. Moderation analyses revealed the interaction between DWMH and hypertension explains 5.85% of the variance in the WAIS score (*P* = 0.0093).

Since Mann–Whitney U-tests and Kendall’s tau correlations found that (i) the relationship between hypertension and WMH was driven by DWMH and (ii) the relationship between cognition and WMH was also driven by DWMH, we focused on DWMH in our main analysis. While controlling for test age, lesion volume, WAB AQ (aphasia severity) and education, the model summary for regression revealed an *R*^2^ of 0.4185 at *P* < 0.0001 (*R* = 0.647, *F* = 7.301) (Table 3). The interaction was statistically significant for hypertension and DWMH (*F =* 7.148, *P* = 0.0093, Table 3). It is important to note in moderation analysis that it is the interaction between the *x*-variable and the moderating variable which is important rather than which variable (hypertension or DWMH severity) is named as the moderator. The interaction shows that the relationship between hypertension and WAIS score is significant at and above a Fazekas score of 2 for the moderating variable, DWMH, see Fig. 4. The overall model explains 41.85% of the variance in WAIS scores (*P* = 0.0001), and the interaction between DWMH and hypertension explains 5.85% of the variance (*P* = 0.0093). The interaction is significant such that the presence of hypertension and higher DWMH scores are together associated with poorer WAIS scores. The interaction became significant at a DWMH score of 2 and was stronger for more severe DWMH. This demonstrates that for people who have a history of hypertension when DWMH severity is higher (≥2), there is a negative relationship between DWMH severity and WAIS scores. Conversely, for individuals with no history of hypertension, DWMH severity was not associated with WAIS scores. Similarly, for those with a history of hypertension, when DWMH severity was low (0 or 1 on the Fazekas scale), there was also no relationship between DWMH severity and WAIS scores.

**Table 3:** Moderation model output

Model summary						
*R*	*R* ^2^	MSE	*F*	df1	df2	*P*
0.6469	0.4185	21.6727	7.3008	7.0000	71.0000	0.0000
Overall model						
	Unstandardized coefficient	Standard error	*t*-value	*P*-value	LLCI	ULCI
*Constant*	−0.0844	5.8632	−0.0144	0.9886	−11.7753	11.6065
*Hypertension*	1.9366	2.1537	0.8992	0.3716	−2.3578	6.2310
*DWMH*	2.6748	1.3164	2.0318	0.0459	0.0499	5.2997
*Lesion volume*	0.0000	0.0000	0.1579	0.8750	0.0000	0.0000
*WAB AQ*	0.0979	0.0280	3.4958	0.0008	0.0421	0.1537
*Test age*	−0.0284	0.0567	−0.5015	0.6176	−0.1414	0.0846
*Education*	0.4575	0.2322	1.9704	0.0527	−0.0055	0.9204
*Interaction*	−4.0379	1.5103	−2.6735	0.0093	−7.0495	−1.026405

Test of higher order unconditional interaction						
	*R* ^2^ change	*F*	df1	df2	*P*	
Hypertension^a^ DWMH	0.585	7.1479	1.0000	71.0000	0.0093	
Conditional effects of the focal predictor at values of the moderator^b^						
	Coefficient	Standard error	*t*-value	*P*-value	LLCI	ULCI
*DWMH: 0*	1.9366	2.1537	0.8992	0.3716	−2.3578	6.2310
*DWMH: 1*	−2.3569	1.1857	−1.9425	0.0560	−4.6675	0.0610
*DWMH: 2*	−6.5431	1.7897	−3.6553	0.005	−9.1762	−2.9745
*DWMH: 3*	−10.1772	2.9575	−3.4411	0.0010	−16.0743	−4.2801

LLCI = lower limit 95% confidence interval.

ULCI = upper limit 95% confidence interval.

df, degrees of freedom; DWMH, deep white matter hyperintensities; LLCI, lower level of the 95% confidence interval; ULCI, upper level of the 95% confidence interval; WAB AQ, Western Aphasia Battery Aphasia Quotient.

^a^The main effects for hypertension and DWMH in the ‘overall model’ section cannot be interpreted since they are part of the interaction.

^b^When calculating the conditional effects of the focal predictor at values of the moderator, the analysis produces the levels of the independent variable, and they cannot be manually decided. Therefore, actual cut-off scores were 0.000, 1.0500, 2.1000 and 3.000. In the table, we have edited to interpretable scores relevant to the scale used to rate DWMH for ease of understanding.

## Discussion

This study aimed to investigate the effect of hypertension on the relationship between WMHs and cognition in people with post-stroke aphasia. We found that participants with a history of hypertension had worse WAIS scores and increased WMH load, particularly DWMH. Then, moderation analyses revealed that DWMHs moderate the relationship between hypertension and WAIS scores (controlling for lesion volume, WAB AQ, education and age). The interaction is significant such that a history of high blood pressure and severe DWMH are associated with poorer WAIS scores. This interaction between DWMH severity and hypertension suggests that, together, overall health and brain health can influence cognition in people with stroke aphasia.

### Hypertension and cognition

Previous research has highlighted the relationship between hypertension and cognitive decline in healthy ageing.^34,35^ Indeed, a review of 58 cross-sectional, longitudinal and randomized placebo-controlled trials found that hypertension was associated with cognitive decline in the majority of longitudinal studies but not all.^36^ The results of our study may explain this inconsistency in the literature, as it is possible that the way in which WMHs, and specifically DWMHs, and hypertension interact may explain cognitive decline rather than hypertension alone. This may be particularly pertinent in stroke survivors where WMHs are common and may affect stroke recovery. Factors known to play a role in the relationship between hypertension and cognitive function include age and education, which were controlled for in our analyses, as well as certain biological qualities of hypertension, which we were unable to control for.^35^ Our findings support the utility of evaluating WMHs as an indicator of brain health when studying cognitive performance in people with hypertension and particularly those with a history of stroke.

### Hypertension and stroke

Apart from its cognitive correlates, hypertension is widely understood to be a major risk factor for stroke, with stroke risk increasing as blood pressure increases.^35,37^ Therefore, many individuals who have experienced a stroke also have a history of hypertension. In addition, Tadic and colleagues found that both hypertension and stroke are independent risk factors for the development of cognitive impairment in older adults.^38^ While stroke has been associated with cognitive decline and an increased likelihood of developing dementia, previous studies have also demonstrated that hypertension is associated with a greater impact of stroke on cognitive function.^39^ Given this, it is possible that the relationship between hypertension and cognitive decline in our participants may be partially explained by individuals with hypertension having more severe strokes, although no difference in mean lesion volume between individuals with and without a history of hypertension was observed in our sample (Table 2).

### Hypertension and white matter hyperintensity severity

Previous research has also demonstrated that hypertension is a risk factor for the development of WMHs in healthy ageing, with one study reporting an association between increasing blood pressure (systolic and diastolic) and increasing total Fazekas scores in hypertensive patients.^37,40^ Furthermore, hypertension has previously been found to be associated with increased WMH volume over time in both stroke-free^41^ and post-stroke populations.^42^ A study of patients with minor ischaemic stroke found that patients with incident cognitive decline 2 years post-stroke demonstrated a higher incidence of WMH progression as compared with those with no cognitive decline. Interestingly, the presence of extensive WMHs at baseline did not predict cognitive decline.^42^ The authors also reported that patients whose WMH volume increased were more likely to have a history of hypertension compared with those whose WMH volume decreased or remained stable during the 2-year follow-up. These findings indicate that the relationship between hypertension and WMH severity may influence cognitive outcomes through an exacerbating effect on existing WMHs in the post-stroke period. Our results support evidence for a relationship between hypertension and WMH severity following stroke and extend previous findings by suggesting that it is DWMH severity rather than PVH severity that drives this relationship in a stroke aphasia population. Conversely, in stroke-free populations previous research has suggested that there is a bigger role of PVH, for example, Zhang and colleagues reported a relationship of PVH with blood pressure variability and mean arterial pressure.^43^ They did also, however, find that blood pressure (both systolic and diastolic) was significantly associated with both PVH and DWMH volumes.^43^ The differential relationship between hypertension and DWMH versus PVH suggests that the location of WMH may be associated with different risk factors in different populations. As many studies combine DWMH and PVH severity into one summary score, our results suggest that it may be useful to consider them separately in future research.

Of note, the moderating effect of DWMH on the relationship between hypertension and WAIS score in our analyses became significant at a Fazekas score of just over 1 point. A DWMH rating of 1 on the Fazekas scale can indicate any number of nonconfluent DWMH and applies to people with even very mild DWMH; there must be confluent WMH in order to elevate the score to 2. As such, the inherent variability of the WMH rating system used likely explains, at least in part, why the interaction effect between hypertension and DWMH severity was not significant for mild DWMHs (score of 1).

There were a small number of outlier participants in our dataset, noticeable in the upper right quadrant of the graph in Fig. 4. It is possible that participants with hypertension who scored highly on the WAIS were those whose hypertension was well-controlled through medical management. In the previously mentioned study of WMH volume changes in survivors of a minor stroke, and the authors found that patients who regularly used antihypertensive medication(s) were more likely to demonstrate WMH regression than stability.^42^ However, clinical trials of antihypertensive medications aimed at preventing or minimizing cognitive decline in stroke and dementia have, to date, provided inconsistent results. Midlife hypertension has become a target for the prevention of later-life cognitive decline, while intensive antihypertensive treatment for very elderly adults may have adverse effects.^38,44^ Future studies could investigate the role of different hypertension management methods on the moderating effect of DWMHs.

The overall model—which included test age, lesion volume, WAB AQ, years of education, DWMH severity, history of hypertension and the interaction between the latter two—explains 41.85% of the overall variation in WAIS score in this group of participants. While the focus of this paper is on hypertension, other cardiovascular risk factors (e.g. diabetes, obesity, hyperlipidaemia) may contribute to the relationship between WMH and cognition on systematic and microvascular levels.^45,46^ The incorporation of factors such as the presence of comorbid conditions, hypertension type and medical treatment have the potential to improve future iterations of the model.^35^

### History of hypertension and white matter hyperintensity severity in stroke aphasia recovery

The interaction between hypertension and DWMH severity and cognition has important implications for recovery in stroke aphasia. While previous research has demonstrated that cognitive decline or regression in recovery progress is common following a stroke, this can be particularly difficult to capture in stroke aphasia where many tests of cognition require language comprehension and production. Therefore, predictors of cognitive decline in stroke aphasia are poorly understood. Here we demonstrate that both cardiovascular health (hypertension) and brain health (DWMH) factors are important predictors of cognition in stroke aphasia, and, critically, the interaction between the two predicts worse cognition. Although our measure of cognition (WAIS) does not completely eliminate the use of language, it minimizes the need to produce language to complete the task, therefore we may be capturing some aspects of cognition which are separate from aphasia. Therefore, these results suggest that cognition in stroke aphasia may be partially influenced by the integrity of the brain tissue spared by the stroke, as well as health variables (hypertension). Given the interaction between DWMH severity and hypertension, these results underscore the importance of preventing hypertension to possibly help preserve cognition in stroke aphasia. Furthermore, the role of successful management of active hypertension in preventing or delaying stroke-related cognitive decline is a topic of current interest in the fields of clinical neurology and neuropsychology.

### Future directions

In addition to exploring other health-related factors, we hope to see if the relationship between hypertension and WMH has a similar effect on speech-language treatment outcomes from the Predicting Outcomes of Language Rehabilitation study. Previous studies have implicated WMH and other markers of cerebrovascular disease in language recovery outcomes in stroke aphasia,^7^ but a model which combines brain health with cardiovascular health markers may be more successful in predicting long-term recovery potential. Regarding cognition, a current study from our group aims to identify factors, including hypertension, which predict susceptibility to cognitive impairment and dementia in survivors of stroke. The study incorporates in-lab measurements of blood pressure as well as a broader array of cognitive assessments.

### Limitations

Limitations of this study include the use of a visual scale (Fazekas) to rate WMHs. As with any visual scale, reliability and uniformity in ratings are subject to variability. This scale does not include the precise location of volumetric information, but the use of four distinct categories aids in ease of use and comprehensibility. The Fazekas scale is commonly used in clinical studies and has been used to help train machine learning models for automatic WMH segmentation.^47,48^ In the present study, we achieved good inter-rater reliability for total, PVH and DWMH Fazekas ratings. It should also be noted that by splitting WMH severity into PVH and DWMH, the variability in severity scores was reduced (i.e. from 0–6 to 0–3). This is a potential explanation for why PVH alone may not have significantly differed between individuals with and without a history of hypertension. Additionally, all participants had aphasia due to a prior stroke, so it is likely that their cognition was affected by these conditions. We endeavoured to control for these factors by adjusting for test age and stroke lesion volume, as well as by using a cognitive measure designed to assess inductive reasoning. Lastly, the hypertension measure was based on self-report, and participants were not asked if they were taking or had previously taken medication for hypertension. A more accurate method of obtaining this information would be to take participants’ blood pressure during study visits and ask participants if they had ever been prescribed medication for hypertension. Therefore, we cannot conclude if it is a history of hypertension or current active hypertension which drives the relationship.

## Conclusion

By investigating the role of both hypertension and DWMHs on cognition in aphasia, we were able to explore the moderating effect of DWMH severity. Importantly, this suggests that health factors and brain health can conjointly influence behaviour. Knowing that hypertension is a modifiable cardiovascular risk factor, a major question raised by our findings is whether successful medical management of hypertension can attenuate the effect of hypertension on cognitive performance after stroke. Clarifying the mechanism by which DWMHs moderate the effect of hypertension on cognition also requires further study. Future studies may also consider the use of volumetric scales to evaluate the relationship of WMH severity and progression with cognition in patients with post-stroke aphasia. The prevalence of hypertension in the global population, particularly among adults over the age of 60, continues to contribute to a decline in quality of life through microvascular changes and major cerebrovascular events. Understanding the relationship among hypertension, brain health and post-stroke cognitive function provides further impetus for the prevention and attentive management of blood pressure at the level of the individual and that of local and national health systems.

## Data Availability

The data that support the findings of this study are available from the corresponding author upon reasonable request.
